# Metabolic dysfunction in human skin: Restoration of mitochondrial integrity and metabolic output by nicotinamide (niacinamide) in primary dermal fibroblasts from older aged donors

**DOI:** 10.1111/acel.13248

**Published:** 2020-09-29

**Authors:** John E. Oblong, Amy Bowman, Holly A. Rovito, Bradley B. Jarrold, Joseph D. Sherrill, Markaisa R. Black, Glyn Nelson, Alexa B. Kimball, Mark A. Birch‐Machin

**Affiliations:** ^1^ The Procter & Gamble Company Cincinnati OH USA; ^2^ Dermatological Sciences Translational and Clinical Research Institute Medical School, Newcastle University Newcastle upon Tyne UK; ^3^ The Bioimaging Unit William Leech Building Newcastle University Newcastle upon Tyne UK; ^4^ Harvard Medical School Boston MA USA

## Abstract

Alterations in metabolism in skin are accelerated by environmental stressors such as solar radiation, leading to premature aging. The impact of aging on mitochondria is of interest given their critical role for metabolic output and the finding that environmental stressors cause lowered energy output, particularly in fibroblasts where damage accumulates. To better understand these metabolic changes with aging, we performed an in‐depth profiling of the expression patterns of dermal genes in face, forearm, and buttock biopsies from females of 20–70 years of age that encode for all subunits comprising complexes I‐V of the mitochondrial electron transport chain. This complements previous preliminary analyses of these changes. “Oxidative phosphorylation” was the top canonical pathway associated with aging in the face, and genes encoding for numerous subunits had decreased expression patterns with age. Investigations on fibroblasts from older aged donors also showed decreased gene expression of numerous subunits from complexes I‐V, oxidative phosphorylation rates, spare respiratory capacity, and mitochondrial number and membrane potential compared to younger cells. Treatment of older fibroblasts with nicotinamide (Nam) restored these measures to younger cell levels. Nam increased complexes I, IV, and V activity and gene expression of representative subunits. Elevated mt‐Keima staining suggests a possible mechanism of action for these restorative effects via mitophagy. Nam also improved mitochondrial number and membrane potential in younger fibroblasts. These findings show there are significant changes in mitochondrial functionality with aging and that Nam treatment can restore bioenergetic efficiency and capacity in older fibroblasts with an amplifying effect in younger cells.

## Graphical abstract

A decline in mitochondrial quality was observed in primary human skin fibroblasts with age, with a decrease in mitochondrial complex gene expression, oxidative phosphorylation rates, spare respiratory capacity, mitochondrial number, membrane potential, and mitophagy. The addition of Nam (nicotinamide, vitamin B3) improved these age‐related changes in cells from older aged individuals to levels observed in younger individuals, with an amplifying effect seen in younger cells.

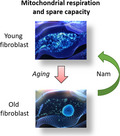

**Abbreviations**
ECAR: Extracellular Acidification Rate
ETC: Electron transport chain
LCM: Laser capture microdissection
MTG: MitoTrackerGreen
MTO: MitoTrackerOrange
Nam: Nicotinamide
OCR: Oxygen Consumption Rate
TMRM: Tetramethylrhodamine Methyl Ester

## 1 INTRODUCTION

The skin is the largest organ of the human body and provides protection from environmental stressors such as solar radiation, industrial pollution, fossil fuel, and carbon emissions. This exposure also makes it highly susceptible to premature accelerated aging due to the cumulative damage caused by these external insults and leads to heightened cellular and structural changes that affect overall functionality, homeostatic state, and appearance. Thus, it is of interest to understand these changes in order to identify mechanistic targets that could prevent premature aging and maintain skin's health and appearance. One of the hallmarks of aging in numerous tissues is alterations in metabolic processes, particularly mitochondrial dysfunction (Ferrucci et al., 2020; López‐Otín et al., 2013; Tonkonogi et al., 2003). This change leads to diminished bioenergetic production as well as shifts and/or reprogramming to compensatory pathways, depending on the cell type and age/disease condition.

To study the effect of aging on metabolism in skin, we analyzed gene expression profiles of biopsies collected from photoexposed upper facial cheek and dorsal forearm and from more photoprotected buttock sites (Kimball et al., 2018). We focused our analysis on the dermal compartment since mitochondrial damage in skin overall has been found to be more prevalent and measurable in dermal fibroblasts, due in part to their more quiescent phenotype as measured by a slower proliferation rate compared to epidermal keratinocytes (Harman, 1972). To better understand these changes at a functional level, we evaluated the impact of age on cellular metabolism using a bank of primary dermal fibroblasts from varying aged donors.

Lastly, nicotinamide (Nam; aka niacinamide, vitamin B_3_) is an essential dietary precursor used in the synthesis of the enzymatic cofactors NAD+ and NADPH (Magni et al., 2004). In addition to maintaining cellular redox status, research has found that NAD+ plays a critical role as a signal transducer in regulating metabolic programming and overall cellular homeostasis (Cantó and Auwerx, 2011). This expanding field of research on the role of these cofactors is showing direct linkages between NAD + metabolism and downstream regulatory points associated with longevity and health (Gomes et al., 2013; Verdin, 2015). One of the original associations between Nam, health, and skin physiology was the discovery that reduced dietary intake of vitamin B_3_ had a direct causative linkage with pellagra (Harris, 1952). Pellagra symptoms include skin dermatitis, dementia, and diarrhea, all of which can be reversed with Nam supplementation. Over the past few decades, Nam has become an important molecule for usage in cosmetic and pharmaceutical products for the treatment of acne, skin photoaging attributes, and skin barrier integrity improvement (Matts et al., 2002; Wohlrab & Kreft, 2014). It has been reported that Nam can protect cells from inflammation, oxidative stress, and metabolic disruption (Sivapirabu et al., 2009; Zhang et al., 2012) and has been found to mitigate some of the acute and chronic damaging effects of UV exposure on skin (Snaidr et al., 2019). Kang et al. (2006) reported that Nam can increase the *in vitro* lifespan of dermal fibroblasts and, subsequently, improve mitochondrial activity (Kang & Hwang, 2009).

Based on this background understanding, we wished to test the impact of Nam on gene expression and mitochondrial activity between young and old aged donor fibroblasts. Since it has been reported that there is a decline in mitochondrial efficiencies in fibroblasts with age (Greco et al., 2003), we wanted to evaluate whether Nam has the potential to impact mitochondrial dysfunction in older aged fibroblasts.

## 2 RESULTS

### 2.1 Oxidative phosphorylation canonical pathway and gene expression of mitochondrial complex subunits are significantly downregulated with age in human dermis

**Figure 1:**
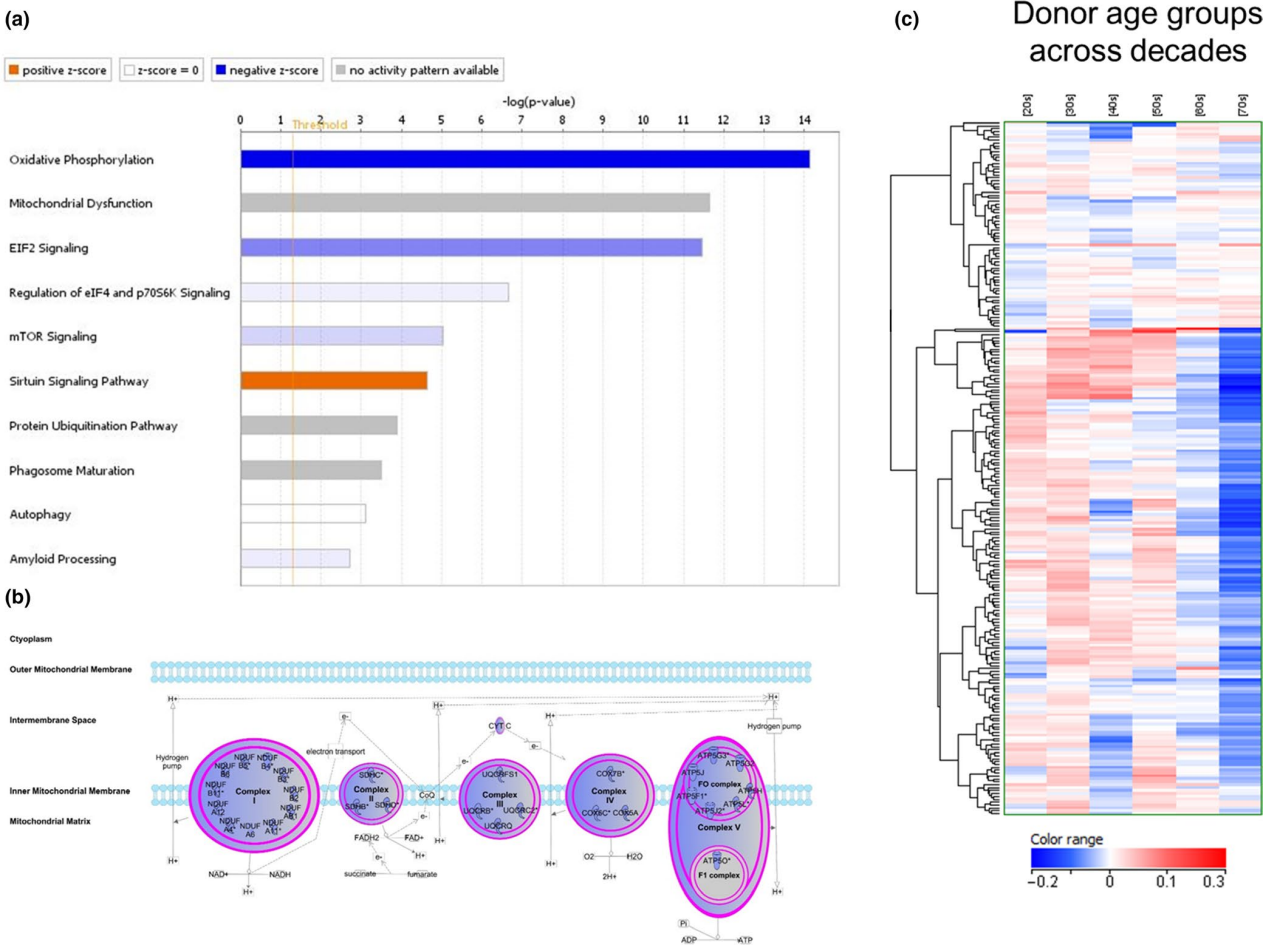
Transcriptomics profiling of dermal sections from female facial cheek biopsies collected across age groups 20–70 years old. (a) Top 10 Canonical pathways in Caucasian face dermis of subject age groups 70 vs 20 (by log *p*) show inhibited oxidative phosphorylation as the top associated pathway (Fisher's *p*‐value = 7.28 × 10^−15^; *z*‐score = −5.92). Graded bar color indicates the magnitude of a pathway's *z*‐score, with predicted activated pathways (*z*‐score >0) shaded in orange and predicted inhibited pathways (*z*‐score <0) shaded in blue. White bars indicate neutral pathways (neither activated nor inhibited) and gray bars indicate pathways in which an activation state cannot be predicted. (b) Oxidative phosphorylation canonical pathway map shows reduced gene expression across all mitochondrial complexes as indicated by blue color. (c) Heatmap of 239 probe sets encoding for subunits of complexes I‐V face dermis from Caucasian subjects across multiple decades (20 s–70 s). Color range reflects normalized intensity values for each age group; blue (lower expression), red (higher expression)

A microarray‐derived transcriptomics data set generated from skin biopsies collected from Caucasian females ranging in age from 20 to 70 (Kimball et al., 2018) was analyzed for canonical pathway changes with age. Of the top ten canonical pathways in the face dermis compartment, comparing 70‐year‐old (*n* = 25) vs 20‐year‐old (*n* = 30) subjects, inhibited “oxidative phosphorylation” was the top associated pathway (Figure 1a). Genes used in the pathway analysis with the most significant fold change between 20‐ and 70‐year olds are listed in Supplemental File S1. To better visualize the changes at the individual subunit level of each complex, an overlay of the gene expression changes in 70‐ vs 20‐year‐old age cohorts onto an oxidative phosphorylation canonical pathway map shows reduced gene expression across all mitochondrial complexes (Figure 1b). A heatmap of 239 probe sets encoding for subunits of complexes I‐V in face dermis samples from Caucasian subjects across multiple decades (20 s–70 s) shows a pattern of significantly reduced expression with aging (Figure 1c).

### 2.2 Gene expression of mitochondrial ETC subunits decreases with age in human dermal fibroblasts as determined via transcriptomics

**Figure 2:**
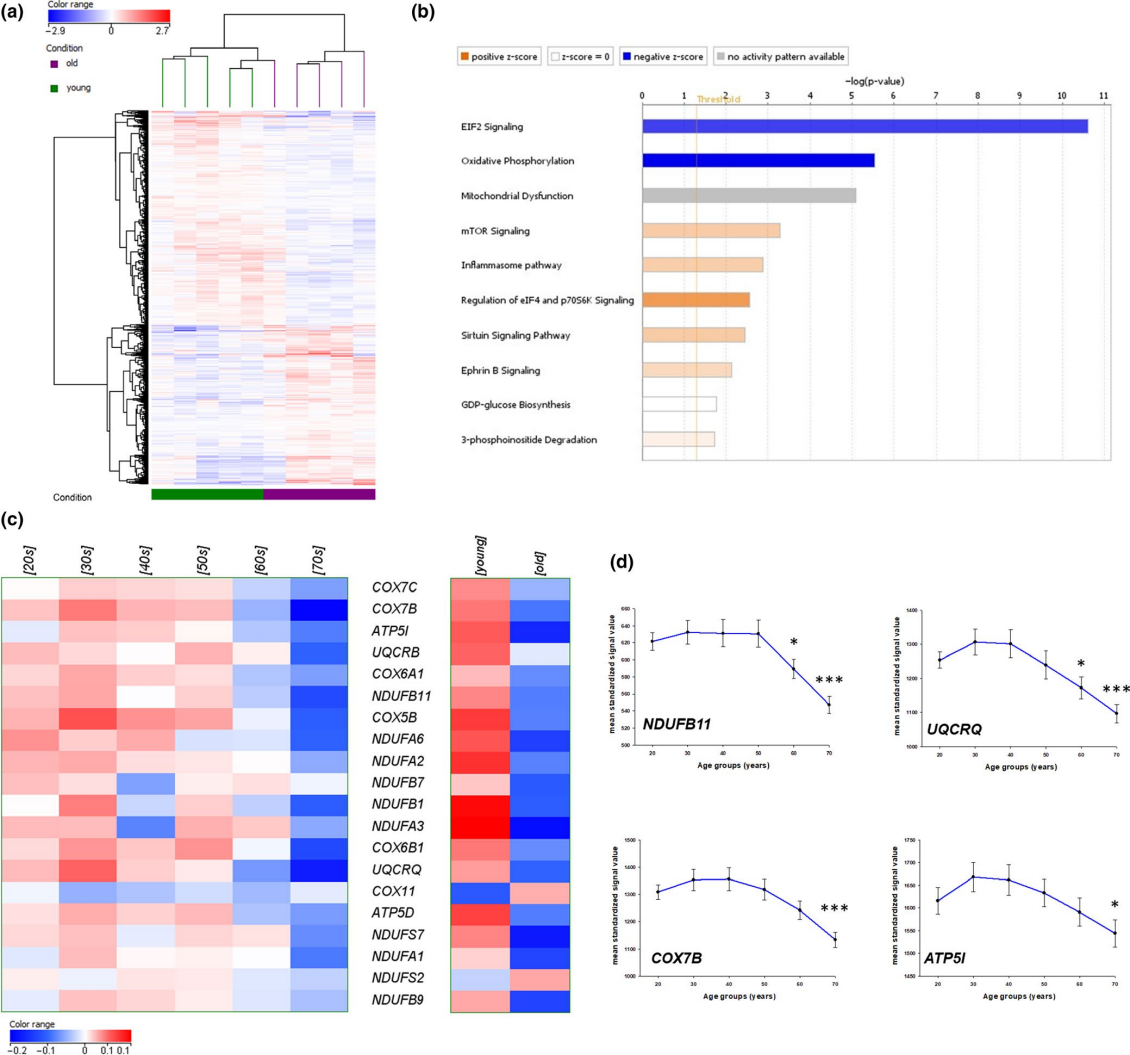
Transcriptomics profiling comparing fibroblasts from younger and older aged donors: (a) hierarchical clustering of the statistically significant probe sets and individual samples in the old (purple, *n* = 5) vs young (green, *n* = 5) fibroblast comparison at day 0. Color range reflects normalized intensity values; blue (lower expression), red (higher expression). (b) Top 10 canonical pathways associated with the old vs. young fibroblast signature at day 0 show inhibited oxidative phosphorylation among top associated pathway (Fisher's *p*‐value = 2.86 × 10^−6^, *z*‐score = 3.58). (c) Heatmap comparing the average relative expression profiles of canonical oxidative phosphorylation genes in dermis biopsy sections from old (70–74 years of age, *n* = 25) and young (20–29 years of age, *n* = 30) subjects (left panel) with old (*n* = 5) vs. young (*n* = 5) fibroblasts (right panel). Color range reflects average normalized intensity values for each group; blue (lower expression), red (higher expression). (d) Trace profiles of probe sets encoding select subunits across age groups from dermal biopsies that were highly downregulated in old fibroblasts compared to young cells. Significance indicates comparisons to 20‐year‐old dermal biopsy group (***p < 0.001, *p < 0.10)

To determine whether these changes in ETC subunit gene expression in aged dermis in vivo could be recapitulated *in vitro* with dermal fibroblasts, we performed microarray analyses on primary human dermal fibroblasts isolated from differently aged donors (Figure 2a). We identified an aged dermal fibroblast signature consisting of 1,677 probe sets differentially expressed between young and old donors. Hierarchical clustering of the aged dermal fibroblast signature revealed consistent clustering among individual samples within their respective age group (Figure 2a). Similar to aged human dermis in Figure 1a, biological pathway analysis showed a significant association of inhibited oxidative phosphorylation canonical pathway with the aged dermal fibroblast signature (Figure 2b). Genes used in the pathway analysis with the most significant fold change between young and older aged donor fibroblasts are listed in Supplemental File S2. Of the 20 downregulated mitochondrial complex genes in aged dermal fibroblasts, 18 were also downregulated in aged dermis in vivo (Figure 2c). Representative probe sets of the most significantly downregulated genes from complexes I, III, IV, and V in the aged dermal fibroblasts showed similar trends across *in vivo* age groups from the dermal section of female facial biopsies (Figure 2d).

### 2.3 Transcript expression levels of mitochondrial and nuclear‐encoded subunits and mitochondrial complex activity increase following Nam treatment

**Figure 3:**
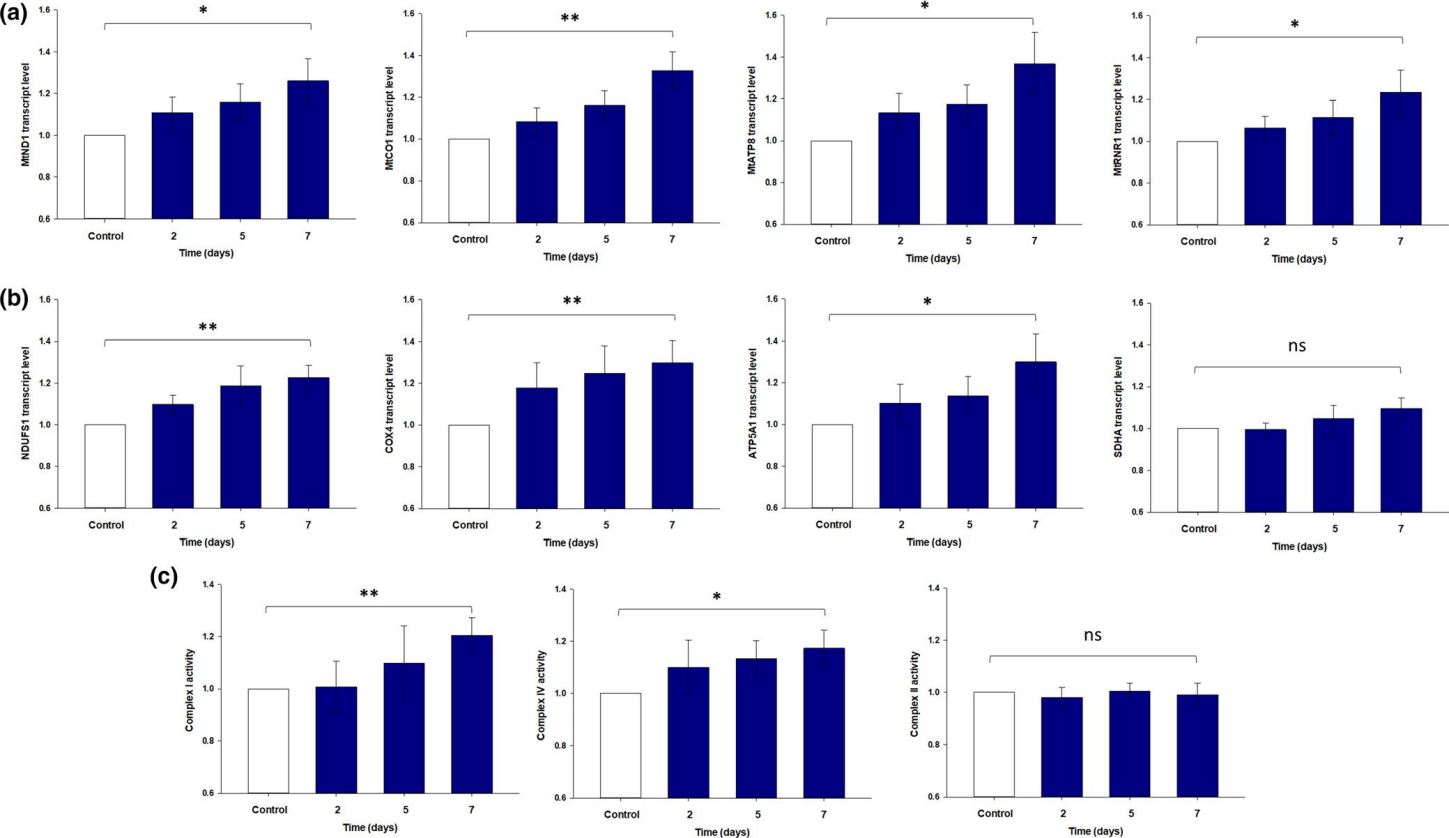
Select transcript expression levels of mitochondrial and nuclear‐encoded subunits following Nam treatment. (a) Following 7 days of Nam treatment, there was a significant increase in gene expression of the mitochondrial‐encoded genes MtND1 (subunit of complex I), MtCO1 (subunit of complex IV), MtATP8 (subunit of Complex V), and the ribosomal gene MtRNR1. As complex II is entirely nuclear‐encoded, no mitochondrial gene expression was able to be measured for this complex. (b) After 7 days of Nam treatment, there was a significant increase in gene expression of the nuclear‐encoded genes NDUFS1 (subunit of complex I), COX4 (subunit of complex IV), and ATP5A1 (subunit of complex V). There was no significant increase in gene expression of SDHA (subunit of complex II). Select complex activity following Nam treatment. (c) Mitochondrial complexes I and IV showed significant increases in activity when measured spectrophotometrically following 7 days of Nam treatment. Measurements of complex II activity showed no change over the 7 days of Nam treatment. The activity of all complexes was normalized against citrate synthase activity, which is used to determine complex activity per unit of mitochondrial content. Experiments were performed using dermal skin fibroblasts obtained from foreskin samples. (*p < 0.05, **p < 0.01, *n* = 13–15 donors ±*SEM*). Nam, nicotinamide; ns, not significant

Primary skin fibroblast cells were treated with 1 mM Nam over a 7‐day period, and gene transcript expression levels were determined for MtND1 of complex I, MtCO1 of complex IV, MtATP8 of complex V, and MtRNR1 for a mitochondrial ribosomal protein. No mtDNA‐encoded subunit for complex II was able to be measured as this complex is entirely nuclear‐encoded. It was observed that expression of MtND1, MtCO1, and MtATP8 was significantly increased following 7 days of Nam treatment (Figure 3a). MtRNR1 also showed a significant increase in expression (Figure 3a), suggesting a general increase in mtDNA copy number (Monnot et al., 2013). Gene transcript levels were normalized to the internal control β‐actin (Li et al., 2011), which did not show a change in expression in response to Nam. These increases in gene expression suggest that long‐term Nam treatment is causing an increase in mtDNA content. We next looked at whether there was correlating increase in the nuclear‐encoded subunits of the mitochondrial complexes. Following the same treatment, we looked for changes in expression of NDUFS1 of complex I, SDHA of complex II, COX4 of complex IV, and ATP5A1 of complex V. There was a significant increase in expression of the complex I subunit NDUFS1 following 7 days of incubation with Nam, as well as for the complex IV subunit COX4 and the complex V subunit ATP5A1 (Figure 3b). However, this was not the case for SDHA, a subunit of complex II, which did not appear to show any significant increase in expression (Figure 3b).

As an increase in gene expression was observed for complexes I and IV, but not complex II, we wanted to determine whether this increase in expression was correlated with an increase in complex activity. The activity of mitochondrial complexes I, II, and IV was measured spectrophotometrically, with the results normalized to citrate synthase activity which is a common mitochondrial marker used to determine mitochondrial amount (Birch‐Machin & Turnbull, 2001). As can be seen in Figure 3c, the activity of complex I increased significantly following 7 days of Nam treatment in primary fibroblasts. The activity of mitochondrial complex IV also showed a significant increase in activity following 7 days of Nam treatment (Figure 3c). However, mitochondrial complex II did not appear to show any change in activity (Figure 3c). These results appear to correlate with the gene expression changes observed earlier. It could be that the increases in gene expression observed for complexes I and IV are resulting in increases in activity and the lack of change observed for complex II gene expression may explain why there was no change in the activity of this complex.

### 2.4 Oxidative phosphorylation and mitochondrial efficiency decline with age in dermal fibroblasts and are restored after treatment with Nam

**Figure 4:**
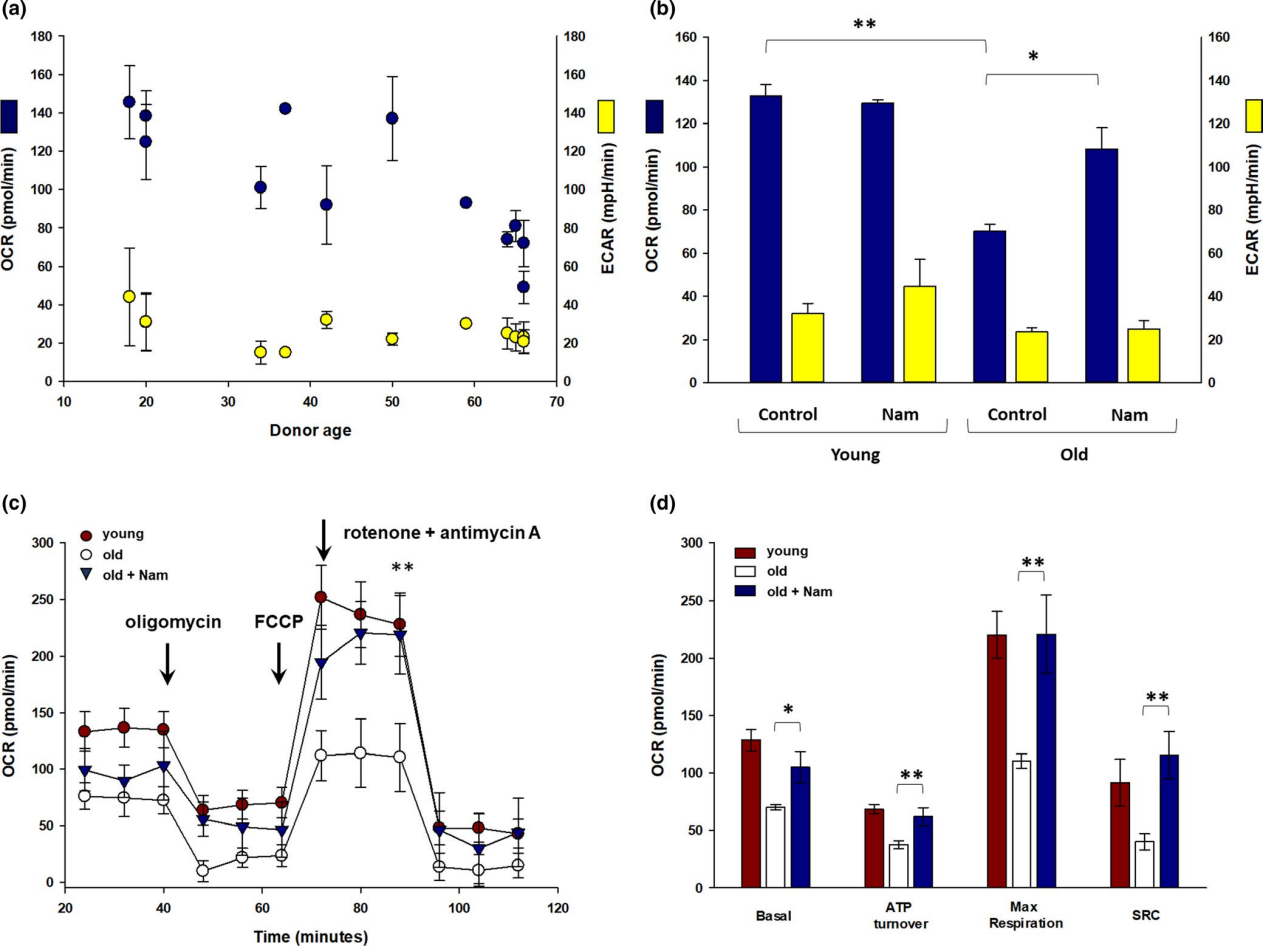
Quantitation of cellular metabolism in primary dermal fibroblasts (ATCC) from females isolated from normal tissue from breast, abdomen, face, or unspecified sites as function of age and effect of Nam treatment on basal metabolism and OCR under stress conditions. (a) Basal measurements of OCR (blue circles) and ECAR (yellow circles) in fibroblasts spanning a range of young to aged donors were quantitated in the Seahorse XF Flux Analyzer. OCR, oxygen consumption rate; ECAR, extracellular acidification rate. (b) Basal measurements of OCR and ECAR in fibroblasts from young (18–20 years of age) and old (64–66 years of age) donors were quantitated. There was a significant difference in basal OCR (blue bars) between young (*n* = 10) and old (*n* = 15). Nam showed a significant increase in basal OCR in old donor cells after incubation for 7 days (*n* = 4). There were no significant differences in basal ECAR (yellow bars) between age groups or after Nam incubation. (c) Fibroblasts from a younger (20 years of age, red circles) and an older aged (65 years of age, open circles) donor were analyzed in the flux analyser and tested with sequential addition of oligomycin, FCCP, and rotenone +antimycin. 7 days of 1 mM Nam treatment of older aged donor cells showed a restoration of OCR to nearly levels in younger aged cells (blue triangles). (d) Quantitation of metabolic output from young (red bars, 18–20 years of age, average of *n* = 2 donors, 3 replicates, ±*SEM*) and older aged (white bars, 64–66 years of age, average of *n* = 3 donors, 5 replicates, ±*SEM*) donor fibroblasts as well as after treatment with Nam for 7 days (blue bars, 64–66 years of age; *n* = 3, ±*SEM*) showed a significant restoration of maximal respiration, ATP turnover and spare respiratory capacity to levels similar to younger aged cells (*p < 0.05, **p < 0.01, ***p < 0.001, ±*SEM*). ECAR, extracellular acidification rate; Nam, nicotinamide; OCR, oxygen consumption rate

To further understand the measured differences in expression of nuclear‐encoded ETC subunit genes between young and old fibroblasts, cells were analyzed in the Seahorse XF Flux Analyzer for oxidative phosphorylation via oxygen consumption rates (OCR) and extracellular acidification rates (ECAR). A comparison of basal OCR values between the 20‐ and 60+ ‐year‐old aged donor range indicated a steady decline of OCR with an apparent inflection point of lower rates after ~50 years (Figure 4a, blue circles). In contrast, there were no significant changes across the age groups in ECAR (Figure 4a, yellow circles).

Since we had previously shown that Nam exposure can protect cellular metabolism under conditions of acute oxidative stress (Rovito & Oblong, 2013), we wished to test whether Nam could also impact chronically changed mitochondrial function in aged fibroblasts. Prior to testing with Nam, we confirmed viability dose ranges in representative primary fibroblasts. To determine toxicity profiles, primary dermal fibroblast cells were incubated with Nam for either 2, 5, or 7 days at concentrations ranging from 0.1 mM to 5 mM. Even at 5 mM Nam over the 7‐day period, there was no change in cell viability (data not shown). This suggests that Nam is not toxic to skin fibroblasts even following long‐term incubation and we chose to use Nam at a concentration of 1 mM in our experiments to maximize any response. A minimum of 3 donors each in the 20‐year‐old range and 60‐year‐old age range group were compared and the average values across repeat experiments and donors showed a 47% decline in OCR values with age when measured at baseline (Figure 4b). Incubating with 1 mM Nam for 7 days showed no significant change in OCR values in younger aged donor cells but a significant increase in older aged donor cells (Figure 4b). There was no significant change in ECAR in either age group.

To understand whether there are any metabolic output potential differences between young and old fibroblasts, metabolic stress testing was performed. Sequential addition of oligomycin, FCCP, and rotenone/antimycin A was used to interrogate ATP turnover rates, spare respiratory capacity (SRC), and maximal oxidative phosphorylation rates. Fibroblasts from older aged donors showed nearly a 50% decline across these measures after stress testing (Figure 4c). Since Nam had a positive effect on restoring oxidative phosphorylation rates in older aged donor fibroblasts, we treated cells for 7 days with 1 mM Nam. Nam treated showed a significant reversal in spare respiratory capacity, ATP turnover, and maximal respiration to measured levels similar to younger aged donor cells (Figure 4d).

### 2.5 Nam increases mitochondrial content and membrane integrity in fibroblasts from both younger and older aged donors

**Figure 5:**
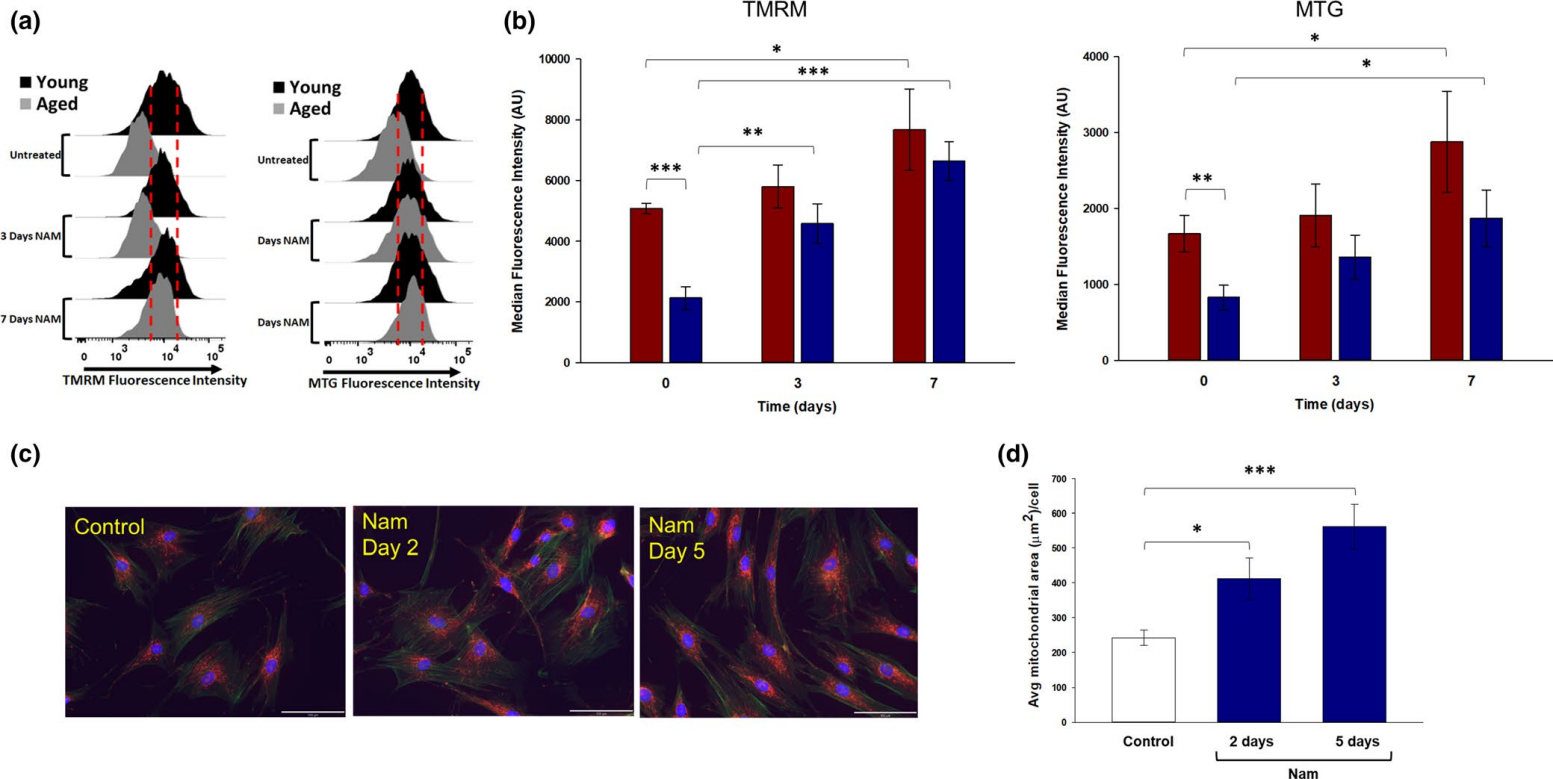
Alteration in mitochondrial integrity and content by Nam in dermal fibroblasts from young and older aged donors. (a) Flow cytometry traces of primary dermal fibroblasts from young and old aged donors incubated with 1 mM Nam and stained with TMRM and MTG at days 3 and 7. Red dotted lines provide visual orientation of shifts across timepoints. (b) Quantification of TMRM and MTG staining intensity of cells from flow cytometry traces show lower mitochondrial integrity and content in older aged fibroblasts compared to young. Treatment with Nam caused significantly higher staining content in both younger (red bars) and older (blue bars) aged donor cells. (c) Fluorescence confocal photomicrographs of older aged donor fibroblasts for MTG (red fluorescence), DAPI for nuclear detection (blue fluorescence), and actin (green fluorescence) after treatment with 1 mM Nam for 2 and 5 days. White bar represents 10 µm. (d) Quantitation of MTO staining in older aged donor fibroblasts after Nam incubation showed a 70% and 132% increase in mitochondrial content area at days 2 and 5, respectively. (*p < 0.05, **p < 0.01, ***p < 0.001 ±*SEM*). MTG, MitoTrackerGreen; MTO, MitoTrackerOrange; Nam, nicotinamide; TMRM, tetramethylrhodamine, methyl ester

Representative fibroblasts from young and old age donors were stained with tetramethylrhodamine, methyl ester (TMRM), and MitoTrackerGreen (MTG) at days 3 and 7 and then submitted to flow cytometry measurements. Staining with TMRM showed a significant difference between young and aged donor cells, suggesting a lowered mitochondrial membrane potential in aged fibroblasts (Figure 5a). Treatment with Nam showed a significant increase in TMRM fluorescence in both young and aged donor cells, suggesting an improved membrane potential. Staining with MTG showed a significant difference between young and old fibroblasts at day 0, indicative of lower mitochondrial content (Figure 5b, TMRM). Aged fibroblasts treated for 7 days with Nam showed a significant increase in MTG fluorescence, suggesting an increase in mitochondrial content (Figure 5b, MTG). Additionally, there was a significant increase in both TMRM and MTG signal in young fibroblasts treated with Nam. Microscopy image capture of fibroblasts stained with MitoTrackerOrange (MTO) showed a significant increase in signal strength as early as after 2 days of treatment with Nam and a further increase in signal after 5 days of Nam treatment (Figure 5c). Quantitation of cellular area occupied by MTO positive staining showed Nam increased MTO signal by 70% at day 2 and 132% at day 5 (Figure 5d).

### 2.6 Nam increases mitophagy in both younger and older aged donor fibroblasts

**Figure 6:**
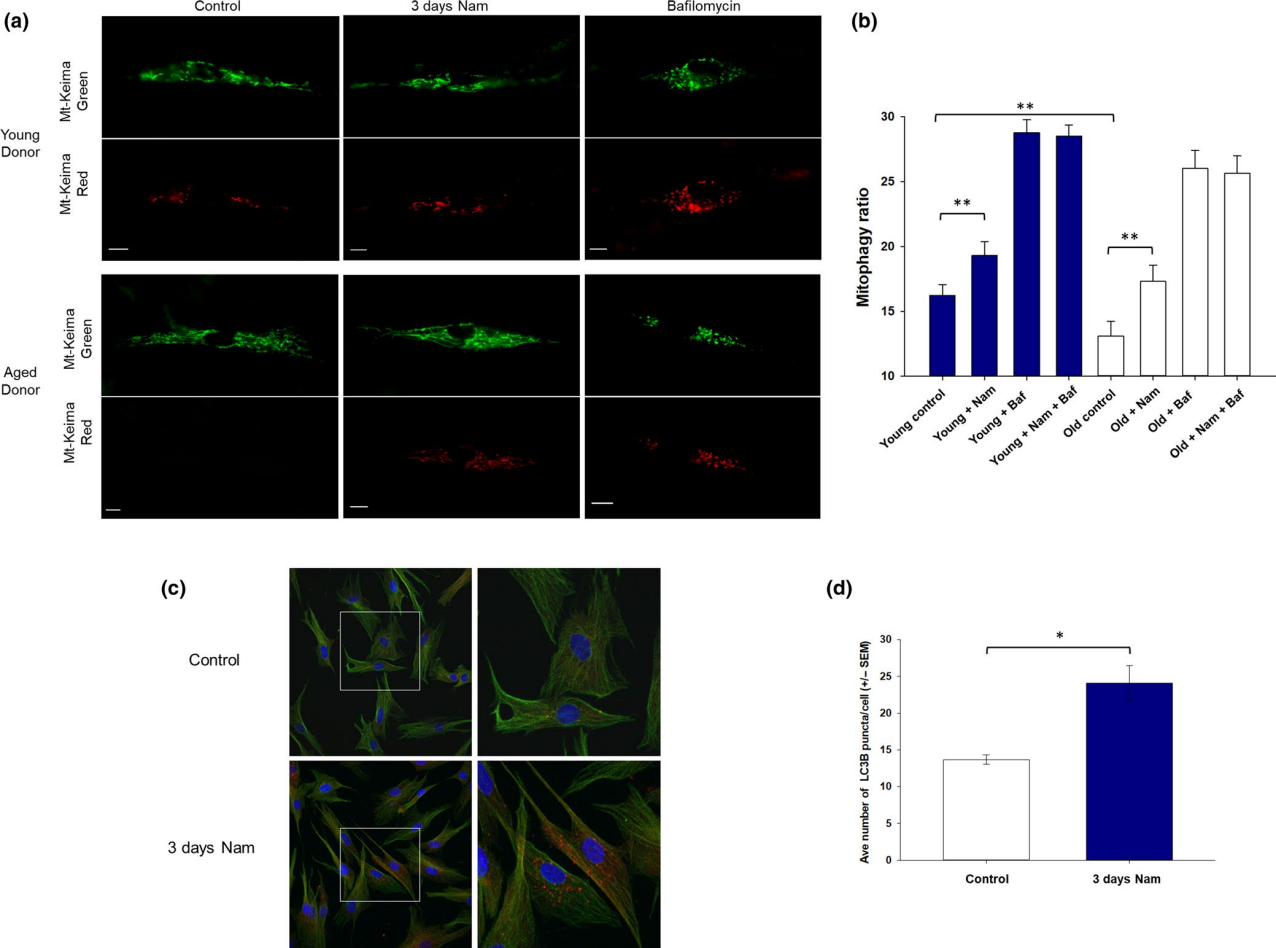
Mitophagy levels following Nam treatment. (a) Example images of human skin fibroblast cells transfected with mt‐Keima showing the mitochondrial network in green and the mitophagic vesicles in red. Cells from a young donor (26 years) and old donor (84 years) showing a control cell, a cell treated with Nam for 3 days, and a cell treated with 0.4 µM bafilomycin. White bar represents 10 µm. (b) Approximately 100–200 cells per category were analyzed (4 donors per category), from either young (17, 26, 28, and 33 years) or aged (75, 80, 83, and 84 years) donors, using dermal skin fibroblasts obtained from foreskin samples. Cells from young donors showed a 23% higher level of mitophagy than aged cells. Cells treated with Nam for 3 days showed a 19% and 32% increase in mitophagy in both younger and older donors, respectively. The bafilomycin controls all showed higher levels of mitophagy. (c) Increased levels of autophagy marker LC3 in dermal fibroblasts with Nam treatment. Fluorescence confocal photomicrographs of older aged donor fibroblasts for LC3 (red fluorescence), DAPI for nuclear detection (blue fluorescence), and tubulin (green fluorescence) after treatment with Nam for 3 days (left panel). Select area from control and Nam‐treated cells at high magnification (right panel) showing the internal distribution of LC3 in puncta form. (d) Number of puncta per cell was quantitated (*n* > 30 cells per treatment group) via image analysis and showed a 73% increase with Nam treatment compared to control cells (***p < 0.001, **p < 0.01, *p < 0.05). Nam, nicotinamide

Mitophagy levels were determined in primary human skin fibroblasts via transfection with the mt‐Keima protein (Katayama et al., 2011). This pH‐sensitive fluorescent protein is targeted to the mitochondria, where it shows pH‐dependent excitation. At a neutral pH (mitochondria not undergoing mitophagy, displayed as green), the excitation is approximately 440 nm, and at a low pH (mitochondria undergoing mitophagy within lysosomes, displayed as red), the excitation is approximately 586 nm (Katayama et al., 2011). Example images of cells from a young and aged donor are shown in Figure 6a, following treatment with Nam for 3 days. We found that the level of mitophagy was significantly higher (by 23%) in skin fibroblast cells from younger individuals as compared to older individuals (Figure 6b). Interestingly, treatment with Nam for 3 days showed a 19% increase in mitophagy levels in younger donor cells (blue bars) and a 32% increase in the older donor cells (white bars). The level of mitophagy in the cells from the older individuals improved following Nam treatment to a level equivalent to that of the cells from the younger individuals (i.e., in the absence of Nam). Treatment with the positive control bafilomycin significantly increased the levels of mitophagy as expected (Figure 6b), as this antibiotic inhibits autophagic flux and therefore prevents mitophagic vesicles from degradation, thus causing their accumulation (Yoshii & Mizushima, 2017).

Fibroblasts from older aged donors were grown with or without Nam for 3 days and then stained for LC3B. Confocal microscopy images of representative fibroblasts from a 65‐year‐old donor showed a consistent pattern of greater number of puncta with Nam treatment compared with control treated cells (Figure 6c). Quantitation confirmed that Nam treatment increased LC3B staining levels by 73% compared to control‐treated cells (Figure 6d).

Our results demonstrate that in photoaged skin, there is a significant reduction in the expression levels of nuclear‐encoded subunits that comprise the complexes of the ETC. As a model of *in vivo* functionality, we used dermal primary fibroblasts from older aged donors to show lower mitochondrial integrity and efficiency. This lowered mitochondrial functionality was shown to be effectively reversed with Nam treatment to the levels measured in fibroblasts from younger aged donors. These findings support the hypothesis that Nam can reverse a hallmark aging process, particularly as measured by cellular bioenergetics restoration in mitochondria in aged skin. This has implications that Nam can prevent and reverse premature skin aging and maintain skin's overall homeostasis state and health (Oblong, 2014).

## 3 DISCUSSION

Like any organ in the human body, skin undergoes aging which can be accelerated by chronic accumulation of damage due to daily exposure to environmental stressors, particularly solar ultraviolet radiation (D'Orazio, Jarrett, Amaro‐Ortiz, & Scott, 2013; Yaar and Gilchrest, 2007). Aging in mammalian cells generally leads to changes in several molecular processes that have been classified as the “Hallmarks of Aging” including mitochondrial dysfunction (López‐Otín et al., 2013). Acute changes in metabolism and cellular bioenergetics due in part to oxidative stress are associated with decreases in mitochondrial function and integrity due to cumulative damage (Birch‐Machin & Bowman, 2016; Bratic & Larsson, 2013; Bratic & Trifunovic, 2010). This dysfunction has significant implications on cellular homeostasis and overall “housekeeping” functions. To better understand any related changes in skin, we evaluated the expression patterns of mitochondrial‐related genes in the epidermal and dermal compartments from laser capture microdissection (LCM) dissected biopsies collected from facial cheek, dorsal forearm, and buttock sites (Kimball et al., 2018). Preliminary analysis by Kimball et al. (2018) suggested that in the epidermis, there is an age‐associated decrease in mitochondrial function as measured by COX7A2L expression. Our work focused on a more in‐depth profiling of gene expression patterns of all subunits comprising complexes I‐V of the ETC in the LCM dermal sections. Canonical pathway analysis identified *oxidative phosphorylation* as the top pathway that is decreased across the decades with age. Supporting this bio‐theme, 18 out of 20 genes encoding for complexes I‐V were found to be significantly downregulated when comparing between 20‐ and 70‐year‐old subjects in facial cheek photoaged biopsies. We observed a similar pattern in the dermal section from dorsal forearm biopsies but not in the more photoprotected buttock biopsies. Interestingly, we also evaluated the LCM dermal sections from an African‐American cohort across 20 s, 40 s, and 60 s and did not observe any significant changes with any of the complex subunit probe sets (data not shown). Separately, we did not see any consistent changes in gene expression in the epidermal sections from any of the biopsy sites. This may be due in part to the more dynamic nature of the epidermis that is continually renewing from the basal layer via proliferation and differentiation (Houben et al., 2007).

We utilized primary dermal fibroblasts from young and older aged donors to evaluate whether similar gene expression changes occur in these cells. A bank of primary dermal fibroblasts from commercial sources and isolated from surgical waste surgery were collated spanning a 20‐ to 70‐year age range. We found that fibroblasts from older aged donors had lowered expression signatures associated with oxidative phosphorylation and key nuclear‐encoded subunits that comprise complexes I, III, IV, and V of the ETC. Notably, the decrease in oxidation phosphorylation predicted by bioinformatic analyses was substantiated through functional assays that showed oxidation phosphorylation rates and overall mitochondrial efficiency are significantly decreased in cells from older aged donors. Interestingly, the bank of primary fibroblasts used in this work was mostly isolated from surgical waste skin removed from more photoprotected sites of stomach, breast, and foreskin. It is possible that the isolated primary cells provided a greater level of sensitivity of measuring changes in gene expression levels as a function of age compared with the LCM isolated dermal fraction from skin biopsies that comprise the total content.

We had previously reported that Nam treatment can protect cellular bioenergetics in dermal fibroblasts from acute oxidative stress with primary effects on ECAR and, to a lesser extent, on oxidative phosphorylation (Rovito & Oblong, 2013). Thus, we asked whether Nam could restore mitochondrial function from chronic induced changes with age with longer exposure periods. Treatment of fibroblasts from older aged donors with Nam over a 5‐ to 7‐day period showed both increases in basal oxidative phosphorylation as well as additional metabolic metrics measured via stress testing and a significant recovery in SRC. Further supporting this metabolic measure is that analysis of expression levels of both mitochondrial and nuclear genes for complexes I and IV was increased following 7 days of Nam treatment. This was also the case for overall mitochondrial complex activity per unit of mitochondria. Flow cytometry staining found that after three days of incubation, Nam had a positive effect on mitochondrial content and integrity in both younger and older aged donor cells. However, treatment with Nam for shorter periods of 1‐2 days did not lead to any of the measured changes in mitochondrial function (data not shown), suggesting that an extended incubation time period was required to allow for these structural changes.

Mitophagy is defined as the selective degradation and recycling of mitochondria by autophagy in order to remove damaged mitochondria and maintain efficient cellular bioenergetics. Of relevance, it has been proposed that strategies to increase mitochondrial activity via complex I will enhance autophagy (Thomas et al., 2018). Mitophagy levels have been reported previously to decline with age in cells from humans and other animals (Diot et al., 2016; Sun et al., 2015). This correlated with our results for which we found higher levels of mitophagy in skin fibroblasts from younger individuals as compared to older individuals. This decline in mitophagy with age is thought to result in an accumulation of dysfunctional mitochondria and therefore decreased cellular efficiency. To evaluate whether Nam treatment had any impact on mitophagy, we used mt‐Keima staining to show that older aged donor fibroblasts had a much lower overall mitophagy index compared with younger donor cells and treatment with Nam for 3 days restored the signal to the levels observed in younger aged donor cells. To further evaluate whether mitophagy is part of Nam's mechanism of action, we stained Nam‐treated older aged fibroblasts for LC3B, a marker of overall autophagy, and found a 73% increase in signal intensity compared to control. In this work, we have demonstrated that incubating cells with Nam shows an increase in mitophagy levels. Our data support that Nam mechanism of action on improving mitochondrial functional activity could include mitophagy induction in not just older aged fibroblasts but younger aged cells as well.

We had previously shown that complex II gene expression and functionality are significantly reduced with age in dermal fibroblasts (Bowman & Birch‐Machin, 2016). However, in the bank of human primary cells used in this study Nam did not have any significant impact on genes encoding complex II subunits nor on complex II functional activity in contrast to complex I, IV, and V. Interestingly, complex II is entirely nuclear‐encoded whereas all the other complex subunits are both nuclear and mitochondrial‐encoded. Thus, it is possible that Nam effects on impacting mitochondrial functionality involve a role of mtDNA‐encoded expression as a necessary event. Lower NAD+ levels can lead to a disruption in nuclear‐mitochondrial communication that occurs during aging (Gomes et al., 2013), and NAD^+^ levels are known to continuously decline with age as measured in skin biopsies across age groups (Massudi et al., 2012). Previous work as well as our own suggests that the mitochondrial ETC complexes that are encoded by both mtDNA and nuclear DNA have increased activity in response to Nam. Thus, it is possible that part of Nam effects is driven via mitochondrial‐nuclear retrograde signaling. It could be speculated that NAM rescues the overall activity of the mitochondria and improves the ETC without requiring a specific improvement in complex II. This could be due to the increased expression and activity of the remaining complexes compensating for a lack of change in complex II, and therefore, no increase in complex II expression is required. Previous work has suggested that a decrease in specific complexes can lead to increases in other complexes as a compensatory method (Baracca et al., 2005; Yen et al., 1996), which may result in differing levels of the individual complexes within the ETC and may explain the lack of change of complex II observed in the current work. Alternatively, it may be possible that the process of mitophagy aids the rescue of complex II and therefore removes the “apparent signal” for increased nuclear expression of complex II subunits, translocation, and assembly. However, more work is needed to better understand the differential effects of Nam on restoring mitochondrial dysfunction that do not appear to be dependent on complex II activity.

The mechanism by which Nam increases mitochondrial complex expression and activity remains to be established but the data presented here suggest that there is an increase in mitophagy activity. It could be hypothesized that the increase in mitochondrial complex activity and expression following Nam treatment in our work could potentially be linked to an increase in mitophagy levels, resulting in higher cellular content of more efficient mitochondria. Mitophagy has been shown previously to be linked to Nam treatment in human fibroblasts (Jang et al., 2012), and a possible increase in mitophagy could explain how mitochondrial activity is able to be maintained, as damaged and inefficient mitochondria are continually being replaced (Melser et al., 2013). It has been shown in the current work as well as in previous studies that both membrane potential and mitophagy are higher in younger individuals and both show a decrease with age, which may seem counter‐intuitive if a low membrane potential results in mitophagy of the mitochondrion. However, it may be that an increase in mitophagy is decreasing the number of mitochondria with low membrane potentials via their removal, since mitophagy limits mitochondrial membrane potential loss as a quality control mechanism (Chen et al., 2020). Those mitochondria with lower membrane potentials may be targeted for degradation via mitophagy, and the increase in degradation of these mitochondria may leave a decreased number of these damaged (low membrane potential) mitochondria present to be measured. The increase in mitophagy seen in young cells or following NAM treatment may therefore correlate with an increase in membrane potential of the remaining mitochondria, without necessarily a causal relationship between both.

We hypothesize that part of Nam's mechanism of action to restore Complex activity and mitochondrial integrity is via mitophagy induction. However, further work is needed to better understand additional Nam effects such as fusion/fission to remove damaged mitochondrial components which is hypothesized to be a process that complements mitophagy (Yoo & Jung, 2018). Additionally, an understanding of Nam metabolism, particularly NAD+ quantitation, is merited along with evaluation of the role of sirtuins and the PGC‐1α pathway as previously shown in a murine aging model (Gomes et al., 2013).

In summary, there is a significant reduction in gene expression of subunits that comprise the mitochondrial ETC in the dermis of photoexposed skin. We used primary dermal fibroblasts that showed similar gene expression differences between younger and older aged donors as a model system to evaluate mitochondrial functional integrity. Treatment with Nam over extended incubation periods led to a nearly complete restoration of mitochondrial quantity and quality to the levels measured in fibroblasts from younger donors. Additionally, we found that Nam's selective effects can be observed as restoring activity of complexes I and IV. Initial data support that induction of mitophagy by Nam exposure could be part of its mechanism of action. Finally, Nam was also found to improve mitochondrial content and integrity in younger aged fibroblasts. These findings add to the growing body of evidence that Nam plays an important role in helping maintain and restore critical cellular functions such as bioenergetics in skin and thereby enhancing skin's resilience, health and appearance.

## 4 EXPERIMENTAL PROCEDURES

### 4.1 Primary cell culture

Primary skin fibroblasts were grown from foreskin samples obtained from donors, following separation of the dermis from the epidermis via dispase treatment. Dermal cells from Caucasian male donors aged between 17 and 84 years old were grown in DMEM containing 10% fetal calf serum and penicillin/streptomycin. Primary human fibroblasts from Caucasian female donors aged between 18 and 66 years old isolated from normal tissue from breast, abdomen, face, and unspecified sites were purchased from American Tissue Culture Collection (ATCC, Manassas, VA) and grown in Eagle's minimum essential medium supplemented with 10% FBS (ATCC) and gentamicin/amphotericin B x500 solution (Invitrogen). To assess cell viability following Nam incubation, a 3‐(4,5‐dimethylthiazol‐2‐yl)‐5‐(3‐carboxymethoxyphenyl)‐2‐(4‐sulfophenyl)‐2H‐tetrazolium (MTS) assay (Promega, UK) was used. Cell viability was assessed in primary dermal fibroblasts from donors aged 46 and 65 years old. Cells were seeded at 5 x 10^3^ cells per well in DMEM to a 96‐well plate, and incubated at 37°C for 16 h. Following incubation, Nam was added to the cells at a range of concentrations from 0.1 mM to 5 mM and cells were incubated for a further 2, 5, or 7 days. After incubation, Nam was removed and the media replaced with DMEM plus MTS, and the cells were incubated at 37°C for 4.5 h. Absorbance was measured at 490 nm using a SpectraMax 250 Microplate Reader and the results viewed using SoftMax Pro V3.1.1.

### 4.2 Gene profiling by microarray

Primary dermal fibroblasts from 5 younger (median age 24 ± 4.8 SD) and 5 older (median age 70.4 ± 15.8) aged Caucasian male donors were subjected to microarray profiling on the GeneTitan U219 array platform (Affymetrix) as previously described (Wu et al., 2018). Microarray profiling from individual face dermis tissue isolated by laser capture microdissection was performed as previously described (Kimball et al., 2018). The cohort in this study was composed of healthy Caucasian females in their 20 s (*n* = 30), 30 s (*n* = 24), 40 s (*n* = 25), 50 s (*n* = 26), 60 s (*n* = 24), and 70 s (*n* = 25). For both the primary dermal fibroblasts and face dermis samples, quartile normalization and Plier summarization were performed on all probe sets. Differentially expressed probe sets were identified using the empirical Bayes method (http://www.stats​ci.org/smyth/​pubs/ebayes.pd). Probe set filtering using a minimum expression threshold (>20th percentile across all samples within any one age group) and hierarchical clustering was performed using GeneSpring version 14.8 (Agilent Technologies, Santa Clara, CA). Data were analyzed through the use of Ingenuity Pathway Analysis version 01‐07 (QIAGEN). Fisher's exact *t* test was used to assess significantly associated pathways, and *z*‐scores were calculated to predict pathway activation states (activated pathways, *z*‐score >0; inhibited pathways *z*‐score <0) as described. Microarray data have been deposited in the Gene Expression Omnibus (GEO) under GSE13​1938 (primary dermal fibroblasts) and GSE11​2660 (face dermis).

### 4.3 Measurement of oxygen consumption and extracellular acidification rates

Oxygen consumption rate (OCR) and extracellular acidification rate (ECAR) measurements from dermal fibroblasts were made using an XF24 Extracellular Flux Analyzer (Agilent/Seahorse Bioscience). Experiments were conducted as described previously (Rovito & Oblong, 2013). Mitochondrial function was assessed by the Mito Stress assay kit (Agilent) using oligomycin, FCCP, rotenone, and antimycin A at final concentrations of 1 µg/ml, 0.25 µM, 1 µM, and 4 µM, respectively. To allow comparison between different experiments, data are expressed as the rate of oxygen consumption in pmol/min or the rate of extracellular acidification in mpH/min, normalized to cell protein in individual wells determined by a Pierce BCA protein assay (Promega).

### 4.4 Measurement of mitochondrial content and mitochondrial membrane potential

Fluorescence‐activated cell sorting (FACS) analysis of mitochondria‐localized fluorescent dyes was used to assess relative mitochondrial content and mitochondrial membrane potential with MTG (ThermoFisher) and TMRM (Invitrogen), respectively (De Luca et al., 2015; Dingley et al., 2012). Propidium iodide (ThermoFisher) was used to detect dead cells. Stained cells were analyzed using a BD LSRFortessa™ cell flow cytometer (BD Bioscience).

### 4.5 Gene expression analysis of mitochondrial subunits

Following Nam treatment, RNA was extracted from skin cells using an RNeasy Mini Kit (Qiagen) and used to generate complementary DNA (cDNA) via reverse transcription using a high‐capacity complementary DNA reverse transcription kit (Applied Biosystems) as per the manufacturer's guidelines. Using the cDNA generated, real‐time quantitative PCR (qPCR) was performed to determine the relative expression levels of the following mitochondrial subunits: MtND1 (143 bp region, complex I, mitochondrial‐encoded), MtCO1 (94 bp region, complex IV, mitochondrial‐encoded), MtATP8 (120 bp region, complex V, mitochondrial‐encoded), MtRNR1 (123 bp region, mitochondrial ribosome, mitochondrial‐encoded), NDUFS1 (80 bp region, complex I, nuclear‐encoded), COX4 (73 bp region, complex IV, nuclear‐encoded), ATP5A1 (117 bp region, complex V, nuclear‐encoded), SDHA (70 bp region, complex II, nuclear‐encoded) (Applied Biosystems). Quantitative PCR was performed by assembling the following components on ice to a final volume of 20 µl per well: deionized H_2_O, 1 × TaqMan Gene Expression Master Mix (Applied Biosystems), 1 × TaqMan Gene Expression Assay primer/probe set (Applied Biosystems), and 20 ng template cDNA. A StepOnePlus real‐time PCR system (Applied Biosystems) was used for the qPCR, with the results viewed using StepOne Software version 2.1 (Applied Biosystems). The following reaction conditions were used: 50°C for 2 min; 95°C for 10 min; and 40 cycles of 95°C for 15 s and 60°C for 1 min. All gene transcript levels were normalized to β‐actin. Relative expression levels were normalized as determined by the 2^–ΔΔ^ *^C^* ^t^ method (Livak & Schmittgen, 2001).

### 4.6 Spectrophotometric analysis of mitochondrial complex activity

Following Nam treatment, skin fibroblasts were analyzed for mitochondrial activity of the individual complexes. The activity levels of citrate synthase, complex I, II, and IV were determined via spectrophotometric methods as described previously (Birch‐Machin et al., 1994).

### 4.7 Measurement of mitophagy using Mt‐Keima

Primary human skin fibroblasts were grown from dermal tissue from 8 individuals categorized as young (<35 years old) or aged (>75 years old). Cells were seeded into 35 mm glass‐bottomed dishes at a concentration of 1 x 10^5^ cells per dish, followed by treatment with 1 mM Nam for 3 days. Cells were transfected with the pH‐dependent fluorescent protein mt‐Keima 48 h before visualization, using Lipofectamine™ 3000 reagent (ThermoFisher Scientific) and Opti‐MEM™ Reduced Serum Medium (ThermoFisher Scientific) according to the manufacturer's instructions. For the positive control, 0.4 µM bafilomycin A1 *Streptomyces griseus* (Merck) was added 1 h before imaging (Dalle Pezze et al., 2014). Live cells were analyzed using a Celldiscoverer 7 (Zeiss) fluorescence microscope. Approximately 40 cells were analyzed per condition per donor. Images were analyzed using Fiji (ImageJ) (Schindelin et al., 2012), for which a threshold was set to remove background autofluorescence, and the level of the green mitochondrial network and the red mitophagic vesicles were measured per cell. The “mitophagy index” showing the percentage of mitochondria undergoing mitophagy per cell was determined by dividing the red area by the red and green areas combined, and multiplying by 100 (Fang et al., 2017). To allow for the best visual representation of the images, the brightness and contrast** **of these images were altered using the best fit function in ZEN 3.1 software (Zeiss) followed by slight manual alteration.

### 4.8 Mitochondria and LC3B fluorescent staining

Primary dermal fibroblasts from aged donors were plated at 1 × 10^4^ per well into µ‐slides (Ibidi, 8 well) in 300 µl of medium per well. Cells were treated with 1 mM niacinamide (Sigma) for 2 and 5 days or 3 and 7 days for Mitotracker Orange and LC3B staining, respectively. Following Nam treatment mitochondria were detected with Mitotracker Orange (Thermofisher). Medium was removed from cells, washed 1× with 300 µl of medium, stained with 300 nM of Mitotracker Orange formulated in medium, and incubated at 37°C, 5% CO_2_, and 90% relative humidity for 30 min. Cells were washed 3× with 1 × PBS, fixed with 200 µl of 4% formaldehyde in PBS and incubated for 15 min at 37°C, washed 2× with PBS and permeabilized in 0.1% Triton X‐100 in PBS, washed and stained with ActinGreen 488 ReadyProbes reagent (Thermofisher), washed and DAPI counter stained using NucBlue fixed cell stain ReadyProbes reagent (Thermofisher). Fluorescent images were captured with a Zeiss Observer.Z1 microscope (Carl Zeiss Microimaging). Relative quantification of the cellular area occupied by MitoTracker positive staining was accomplished via Image Pro Premier™ (Media Cybernetics MD) software utilizing red channel images acquired at equal gamma values, pixel range and exposure. Following Nam treatment LC3B was detected as follows: cells were washed in PBS and fixed with ice cold methanol, permeabilized in 0.1% Triton X‐100 (Thermofisher) in PBS, and blocked for 1 h with 3% BSA in PBS. Cells were incubated overnight at 4°C with a LC3B primary (Abcam 192890, 1:500), diluted in PBS containing an Alexa Fluor 488 conjugated Alpha Tubulin antibody (Abcam 195887, 1:250). Cells were washed in PBS and stained with AlexaFluor 555 secondary antibodies (Abcam 150086, 1:1000) in PBS for 1 h at room temperature, washed and DAPI counter stained using NucBlue fixed cell stain ReadyProbes reagent (Thermofisher). Fluorescent images were captured with a Nikon A1 LUN‐V Inverted confocal microscope. Image Pro Premier™ (Media Cybernetics MD) software and max projection red channel images were utilized to quantitate the number of the LC3B puncta per cell.

### 4.9 Statistical analysis

For the MTS assay, one‐way analysis of variance, ANOVA, with Dunnett's test was used. When comparing differences between untreated control groups and Nam treated groups, an unpaired *t* test was used. For comparison between the untreated control sample and the range of conditions, Statistical analysis was performed using GraphPad Prism 7 (GraphPad Software, USA).

## CONFLICT OF INTEREST

J.E.O., J.D.S., B.B.J., M.R.B., and H.A.R. are full‐time employees of The Procter & Gamble Company (Cincinnati, OH, USA). All other authors declare no conflicts of interest.

## AUTHOR CONTRIBUTIONS

J.E.O. and M.B.M. primarily designed the study and experiments; A.B., H.A.R., M.R.B., B.B.J., and G.N. performed the experiments and analyzed the data; J.D.S contributed materials/analytic tools; J.E.O. wrote the manuscript; M.B.M., A.B., H.A.R., and A.B.K. reviewed the manuscript; All authors read and approved the final manuscript.

## ETHICAL APPROVAL

Primary skin fibroblasts were grown from foreskin samples obtained from donors from the Royal Victoria Infirmary (Newcastle upon Tyne, UK). Ethical approval for primary dermal foreskin work was granted by the Newcastle and North Tyneside Research Ethics Committee (Ref 08/H0906/95), and the research use of the samples was in accordance with the terms of the written, informed patient consent. The study was performed in accordance with the principles of the Declaration of Helsinki.

## Supporting information

File S1

File S2

## Data Availability

The data reported in this paper have been deposited in the Gene Expression Omnibus (GEO) database, https://www.ncbi.nlm.nih.gov/geo (accession no. GSE11​2660). No other datasets were generated or analyzed for this manuscript.
